# Human Anti-Aβ IgGs Target Conformational Epitopes on Synthetic Dimer Assemblies and the AD Brain-Derived Peptide

**DOI:** 10.1371/journal.pone.0050317

**Published:** 2012-11-27

**Authors:** Alfred T. Welzel, Angela D. Williams, Helen P. McWilliams-Koeppen, Luis Acero, Alfred Weber, Veronika Blinder, Alex Mably, Sebastian Bunk, Corinna Hermann, Michael A. Farrell, Hartmut J. Ehrlich, Hans P. Schwarz, Dominic M. Walsh, Alan Solomon, Brian O’Nuallain

**Affiliations:** 1 The Conway Institute, University College Dublin, Belfield, Dublin, Republic of Ireland; 2 Human Immunology and Cancer Program, Department of Medicine, University of Tennessee Graduate School of Medicine, Knoxville, Tennessee, United States of America; 3 Baxter BioScience, Vienna, Austria; 4 The Laboratory of Neurodegenerative Research, Brigham and Women’s Hospital, Harvard Institutes of Medicine, Boston, Massachusetts, United States of America; 5 Dublin Brain Bank, Pathology Department, Beaumont Hospital, Dublin, Ireland; Stanford University School of Medicine, United States of America

## Abstract

Soluble non-fibrillar assemblies of amyloid-beta (Aβ) and aggregated tau protein are the proximate synaptotoxic species associated with Alzheimer’s disease (AD). Anti-Aβ immunotherapy is a promising and advanced therapeutic strategy, but the precise Aβ species to target is not yet known. Previously, we and others have shown that natural human IgGs (NAbs) target diverse Aβ conformers and have therapeutic potential. We now demonstrate that these antibodies bound with nM avidity to conformational epitopes on plate-immobilized synthetic Aβ dimer assemblies, including synaptotoxic protofibrils, and targeted these conformers in solution. Importantly, NAbs also recognized Aβ extracted from the water-soluble phase of human AD brain, including species that migrated on denaturing PAGE as SDS-stable dimers. The critical reliance on Aβ’s conformational state for NAb binding, and not a linear sequence epitope, was confirmed by the antibody’s nM reactivity with plate-immobilized protofibrills, and weak uM binding to synthetic Aβ monomers and peptide fragments. The antibody’s lack of reactivity against a linear sequence epitope was confirmed by our ability to isolate anti-Aβ NAbs from intravenous immunoglobulin using affinity matrices, immunoglobulin light chain fibrils and Cibacron blue, which had no sequence similarity with the peptide. These findings suggest that further investigations on the molecular basis and the therapeutic/diagnostic potential of anti-Aβ NAbs are warranted.

## Introduction

Alzheimer’s disease (AD) is a devastatingly common age-related disorder that progressively affects regions of the brain that are associated with higher cognitive functions, such as memory and learning [1]. Pathological hallmarks of AD include, extracellular neuritic deposits of fibrillar Aβ, a 38–43 peptide fragment of the amyloid precursor protein, intraneuronal neurofibrillary tangles of hyperphosphorylated tau protein, synaptic loss and cortical atrophy [2]. Increasing experimental evidence indicates that soluble Aβ aggregates are an upstream pathological species that may cause neuronal compromise through a variety of different not mutually exclusive mechanisms, including toxicity that is associated with prion protein, and which occurs in synergy with hyperphosphorylated tau [3], [4].

Although current therapy for AD is only palliative, extensive *in vitro* and *in vivo* studies indicate that anti-Aβ immunotherapy may potentiate the disease for some patients [2], [5], [6]. However, results have been mixed for recent clinical trials that focused on passive or active vaccination of anti-Aβ antibodies for AD [5], [6]. Presumably this is because the disease was treated at an advanced stage and/or it is not clear if these antibodies targeted the most pathogenic Aβ species. Nevertheless, anti-Aβ therapy remains one of the most promising approaches for AD, and a reagent that slows disease progression by about a decade may be sufficient to reduce its incidence by a third [7]. To-date, most passive immunotherapy trials have used humanized anti-Aβ monoclonal antibodies that recognize linear sequence epitopes, and react with multiple Aβ conformers, including native non-pathogenic monomers. An alternative therapeutic approach is to supplement a patient’s naturally occurring antibody (NAb) response against pathogenic Aβ since these antibodies may decrease with aging and AD [8], [9]. To-date, four open-label clinical trials, phase’s I-II, have been completed on mild to moderate AD patients treated with intravenous immunoglobulin (IVIg; commercially available preparations that consist of a broad spectrum of polyclonal human IgGs purified from 1000’s of normal individuals, including anti-Aβ NAbs [10], [11]) [12], [13]. Although these studies involved small patient numbers and lacked control groups, the results indicate that IVIg was well tolerated, and benefitted some patients by slowing memory decline [12], [13]. Given these promising results, two randomized, placebo-controlled phase III and a phase II studies on IVIg for AD are ongoing [12].

Anti-Aβ NAbs mechanism of action on AD is not yet known, and it is possible that in addition to anti-Aβ NAbs, other antibodies, such as anti-inflammatory and immune modulatory IgGs, may act in synergy to benefit an AD patient [14], [15]. Nevertheless, results from recent clinical trials have demonstrated IVIg’s ability to reduce an AD patient’s soluble pool of cerebral Aβ and increase the amount of Aβ in the blood [12], [13] – a process consistent with the beneficial effect of anti-Aβ immunotherapy [5], [12], [13]. Extensive *in vitro* and transgenic mice studies have indicated that anti-Aβ NAbs have therapeutic potential for AD and have multiple mechanisms of action [11], [12], [16], [17], [18], [19], [20], including an ability to neutralize toxic Aβ species [8], [18], [21]. These antibodies can specifically react with a plethora of synthetic Aβ conformers [11], [17], [18], [19], and cross-react with non-Aβ amyloidogenic aggregates [17], [22]. However, it is unclear what portion of their Aβ reactivity is against synaptotoxic Aβ assemblies, and if NAbs have a preference for conformational [23] or linear sequence [24] epitopes. An antibody that recognizes a conformational epitope on synaptotoxic Aβ conformers would have clear therapeutic advantage to an antibody that indiscriminately recognizes a linear sequence epitope on all Aβ species, including physiologically relevant monomeric peptide. Although the precise Aβ species to target is not yet known, substantial experimental evidence suggests that soluble non-fibrillar Aβ aggregates are the proximate synaptotoxic species [25], [26], [27], [28]. Given our current poor understanding on how anti-Aβ NAbs function, we are focused on deciphering NAb’s specificity for pathogenically relevant Aβ conformers, and how these interactions may modulate Aβ’s synaptotoxicity. Here we now report investigations on NAb’s specificity for conformational and linear sequence Aβ epitopes, synthetic Aβ dimer assemblies, and AD brain-derived Aβ. Our findings strongly indicate that further investigations on the diagnostic/therapeutic relevance of NAb-Aβ interactions are warranted.

## Materials and Methods

### Ethics Statement

Human brain tissue was obtained from the Dublin Brain Bank (www.brainbank.ie). The brain bank has written informed consent from patients for all tissue samples, and the collection and processing of brain tissue was approved by the Royal College of Surgeon’s in Ireland (RCSI) Ethics Committee. The human brain tissue samples were received and used in accordance with University College Dublin’s Human Research Ethics Committee guidelines (under approval LS-E-10-10-Walsh).

### Proteins, Peptides, and Chemicals

Wild-type human Aβ(1–40) (Aβ), DAEFRHDSGY-EVHHQKLVFF-AEDVGSNKGA-IIGLMVGGVV, Aβ in which serine 26 was substituted with cysteine, and Aβ that contained a N- or C-terminal cysteine and phenylalanine instead of proline at position 19 were synthesized and purified by Dr. James I Elliott at Yale University (New Haven, CT). Peptide mass and purity (>95%) were confirmed by electrospray ionization/ion trap mass spectrometry and reverse-phase HPLC. Custom-made overlapping Aβ peptide fragments (a Pepset™ library) were purchased crude from Mimotopes (Raleigh, NC, USA), and masses confirmed by electrospray mass spectrometry. An immunoglobulin light chain (LC) variable domain λ_6_ protein, Jto, was produced in *Escherichia coli* and purified as described previously [29]. An anti-Aβ N-terminal mAb, 6E10, was purchased from Signet (Dedham, MA, USA). A polyclonal rabbit anti-Aβ antibody, AW8, was raised against aggregated synthetic Aβ(1–42) and recognizes multiple linear epitopes and can bind to a variety of Aβ conformers [28]. Intravenous immunoglobulin (IVIg; Gammagard liquid®) was provided by Baxter BioScience (Vienna, Austria). ImmunoPure Horseradish peroxidase (HRP) and H_2_O_2_ (30% in water) were from Pierce (ThermoFisher Scientific, Waltham, MA, USA). Unbranched dextran standards of molecular masses: 43,800; 21,400; 9890, and 4440 were purchased from Pharmacosmos (Holbaek, Denmark). All other chemicals were obtained from Sigma-Aldrich (Saint Louis, MI, USA) and were of the highest purity available.

### Preparation of Amyloidogenic Conformers

WT Aβ monomers, dityrosine cross-linked Aβ protein species (CAPS), disulfide cross-linked S26C Aβ dimers ([S26CAβ]_2_), protofibrils formed from [S26CAβ]_2_ (PFs), and Aβ and LC fibrils were generated as described below, and were used immediately or frozen at −80°C. WT and S26C peptide concentrations were determined by absorbance at 275 nm using the molar extinction coefficient for tyrosine at 275 nm (ε_275_ = 1400 M^−1^.cm^−1^). The concentrations of CAPS and LC protein were determined using the MicroBCA assay (ThermoFisher Scientific) with a BSA standard curve.

WT and S26C Aβ monomers and [S26CAβ]_2_ were isolated from different sized Aβ assemblies using size exclusion chromatography (SEC). Peptides were incubated in 50 mM Tris-HCl containing 6 M guanidine HCl, pH 8.0, to dissociate pre-existing aggregates, and then characterized on a HiLoad 16/60 Superdex™ 75 column (GE Healthcare Bio-Sciences AB, Uppsala Sweden) eluted at 0.8 ml/min in 25 mM ammonium acetate, pH 8.5 [30]. Fractions that contained Aβ monomers or [S26CAβ]_2_ were analyzed by SDS-PAGE using 16% polyacrylamide tris-tricine gels and silver staining [31]. Meta-stable Thioflavin T (ThT) positive PFs were generated by diluting freshly-isolated [Aβ40S26C]_2_ to ∼0.1 mg/ml in 20 mM sodium phosphate, pH 7.4, and incubating the reaction mixture for ∼3 d at 37°C [30]. The reaction was monitored by ThT fluorescence and was judged complete when the fluorescent signal reached a maximum plateau value [32] (Fig. S1A). Formation of protofibrils was confirmed by electron microscopy (EM), and by the presence of ThT positive aggregates in reaction supernatants after centrifugation at 16,000×g for 20 min (Figs. S1B-D & S3).

ThT positive CAPS were generated by incubating ∼0.2 mg/ml Aβ for ∼3 h at 37°C with 1.1 µM HRP and 250 µM H_2_O_2_ in PBS, pH 7.4, and the reaction product purified using copper (CuSO_4_) precipitation [11]. Dityrosine cross-linked peptide was confirmed by SDS-PAGE, ThT and dityrosine fluorescence emission spectra (Figs. S2 & S3). Fluorescence emission was determined with excitation at 320 nm and emission at 350–550 nm, using an Aminco Bowman series 2 spectrofluorimeter.

WT Aβ and LC fibrils were generated by incubating 0.2 mg/ml of the amyloidogenic proteins in PBS containing 0.02% sodium azide, pH 7.4, at 37°C for 14 days [23]. Fibrillogenesis was judged complete when Thioflavin T (ThT) fluorescence reached maximum plateau values. The reaction products were harvested by centrifugation at 20,200×g for 30 min at room temperature, and fibril morphology confirmed by negative contrast EM (Fig. S2).

### TBS Extract of Human Brain

Frozen temporal cortices from 54 and 66 year-old females each with dementia, fulminant amyloid and tangle pathology (Braak stage = ×and y, respectively) were obtained from the Dublin Brain Bank (www.brainbank.ie). Tris-buffered saline (TBS) extracts of AD brain specimens were prepared, as described previously, divided into 0.3 mL aliquots, and stored at –80°C [28]. The presence of Aβ in each TBS extract was confirmed using a highly sensitive immunoprecipitation/western blotting protocol and the peptide’s concentration estimated by comparison with synthetic Aβ standards [28].

### Isolation of Anti-Aβ NAbs

Aβ-reactive NAbs were isolated from IVIg using Aβ conformer, LC fibril, or Cibacron blue affinity chromatography [11], [17]. Aβ fibril-, CAPS-, or LC fibril-isolated antibodies were generated by passing, at a flow rate of 1 ml/min, 50 ml of 0.22 µm filtered 10 mg/ml IVIg in binding buffer (PBS, pH 7.4) through a fibril or CAPS column consisting of the amyloidogenic conformer cross-linked to N-hydroxysuccinimide (NHS) Sepharose®4 fast-flow agarose matrix (Amersham Biosciences Corp., Piscataway, NJ). Aβ monomer-isolated antibodies were generated using an affinity column consisting of an equimolar mix of monomeric N- or C-terminal cysteine containing F19P Aβ cross-linked to iodoacetyl coupling gel (SulfoLink coupling resin, Pierce) [11]. The antibody flow through was collected, and the column washed with binding buffer until the wash solution had no appreciable absorbance at 280 nm. Fibril-bound IgGs were eluted in 1-mL fractions using 0.1 M Glycine-HCl, pH 2.7, into Eppendorf tubes containing neutralization buffer (1 M Tris-HCl, pH 9.0). The concentration of antibody in the eluant and flow through fractions was determined by absorbance at 280 nm using an extinction coefficient of 1.25 and a relative molecular mass of 150,000. Antibody fractions were pooled, buffer exchanged into PBS and concentrated to ∼1 mg/ml using a PL-30 Centricon (Millipore) apparatus, used immediately or stored at −80°C.

Cibacron blue F3GA-isolated IgGs were generated using an Affi-Gel Blue gel column (Bio-Rad Laboratories, Hercules, CA). Briefly, 50 ml of 0.22 µm filtered 20 mg/ml IVIg in binding buffer (PBS containing 0.6 M NaCl, pH 7.4) was passed through the dye column at a flow rate of ∼1 ml/min. The column was washed with binding buffer until there was no absorbance at 280 nm, and the dye-bound antibodies eluted using high salt buffer (PBS containing 1.5 M NaCl, pH 7.4). As described above, the concentration of antibody in column fractions was determined by absorbance at 280 nm, and the antibody preparations were pooled, concentrated to 1 mg/ml, and buffer exchanged into PBS.

### Antibody Binding to Amyloidogenic Conformers

Our standard ELISAs [33], europium-linked immunosorbant microtiter plate assay [23], and immunoprecipitation (IP)/Western blot protocol [34] were used to establish NAb and control antibody’s interactions with synthetic Aβ conformers, LC fibrils, and AD brain-derived Aβ. Antibody binding to microtiter plate-immobilized amyloidogenic conformers (400 ng/well) was carried out in the presence or absence of solution-phase Aβ inhibitors in assay buffer consisting of PBS containing 1% BSA and 0.05% Tween 20, pH 7.4. A biotinylated polyclonal goat anti-human or anti-mouse IgG (μ heavy chain specific; Jackson ImmunoResearch Laboratories, Inc., West Grove, PA, USA) served as the secondary antibody. Streptavidin-HRP (Jackson ImmunoResearch Laboratories, Inc.) and TMB substrate (SureBlue Reserve™; KPL, Gaithersburg, MD, USA), or streptavidin-europium and enhancement solution (DELFIA®; PerkinElmer, Inc., Boston, MA, USA) served as the detection system. Antibody binding to solution-phase amyloidogenic conformers was determined using a capture ELISA consisting of plate-immobilized antibody (∼400 ng/well) binding to synthetic Aβ conformers in assay buffer. A polyclonal rabbit anti-Aβ antibody, AW8 [28] served as the secondary antibody, and a HRP-conjugated donkey anti-rabbit IgG (whole molecule, GE Heathcare, Buckinghamshire, UK) and TMB substrate (SureBlue Reserve™; KPL) were used as the detection system.

Immunoprecipitation/Western blot analysis of solution-phase antibody binding to synthetic Aβ conformers and AD brain-derived Aβ was investigated using samples that were precleared for nonspecific protein binding to the antibody capture beads (a 1∶1 mixture of Protein A sepharose (Sigma) and Protein G agarose (Roche, Mannheim, Germany)) [34]. Briefly, 0.5 ml of neat TBS extract of AD or normal brain, or the same volume of a synthetic Aβ conformer (diluted to ∼0.2–20 µg/ml with 1% BSA in PBS containing 0.05% Tween, pH 7.4), was incubated with 20 µl of antibody capture beads for 1 h at room temperature. The beads were sedimented by centrifugation at 4,000×g for 5 min, the supernatant collected, IVIg (100 µg/ml) or AW8 (200 µg/ml) added to the precleared samples, and the mixtures incubated as before. The antibody-bead complexes were sedimented, supernatants removed, pellets washed, and antibody-bound Aβ liberated by heating the pellet at 100°C for 5 min in 2×SDS sample buffer [34]. The boiled samples were immediately electrophoresed on 16% polyacrylamide tris-tricine gels, transferred onto 0.2 µm nitrocellulose (Optitran, Schleicher and Schull, Germany) at 400 mA for 2 h. Membrane-bound Aβ was then detected using an equimolar mixture (0.5 µg/ml each) of N-terminal and mid-region Aβ-reactive mAbs 6E10 and 4G8, and enhanced chemiluminescence (Thermo Fisher Scientific Inc, Rockford, IL) as the detection system [34].

### Thioflavin T Fluorescence

Fluorescent measurements were carried out in duplicate by diluting an Aβ or LC sample to 3 µg/100 µl/well with assay buffer (PBS, pH 7.4, or 25 mM ammonium acetate, pH 8.5, containing 30 µM ThT) in a black polystyrene 96-well microtiter plate (ThermoFisher Scientific). ThT signal was determined with excitation at 435 nm and emission at 485 nm using a SpectraMax M2 multi-detection microplate reader (Molecular Devices Corp., Sunnyvale, CA) [35].

### Electron Microscopy

Negative contrast EM was performed by applying 10 µl aliquots of a test sample on to duplicate carbon-coated formvar grids (Electron Microscope Sciences, Washington, PA) that were cross-linked with 0.5% (v/v) glutaraldehyde, and stained with 2% (w/v) uranyl acetate solution (Ted Pella, Inc., Redding, CA). The EM grids were examined using a Tecnai™ G^2^ Spirit BioTWIN electron microscope (FEI, Hillsboro, OR).

### Circular Dichroism Spectroscopy

Aβ solutions (∼0.2 mg/ml) were placed in a 1 mm path length quartz cuvette (Starna Scientific Ltd., Essex, UK) and spectra obtained at 22°C using a J-810 JASCO spectropolarimeter (JASCO Corp., Tokyo, Japan) [30]. Spectra were generated from three data accumulations between ∼195–260 nm with 10 nm/min continuous scanning and a 0.5 nm bandwidth. Raw data were manipulated by subtraction of buffer spectra and by binomial smoothing according to the manufacturer’s instructions (JASCO Corp.).

### General Curve Fitting

Antibody binding curves and aggregation time courses were fitted using SigmaPlot 2000, version 6 (Systat Software, Chicago, IL) or GraphPad Prism, version 5 (GraphPad Software, Inc., La Jolla, CA, USA).

## Results

### NAbs Primarily Recognize Aβ-related Conformational Epitope(s)

**Figure 1:**
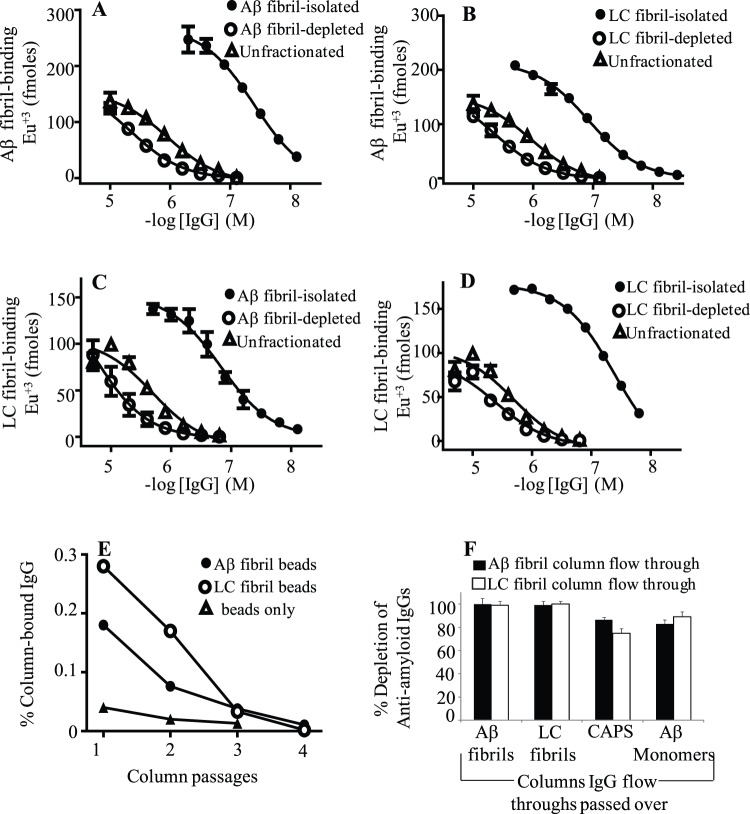
Aβ and LC fibril fractionated NAbs binding to amyloid fibrils. The antibody binding curves are for Aβ fibril (**A**) and LC fibril (**B**) fractionated IVIg against plate-immobilized Aβ fibrils, and for Aβ fibril- (**C**) and LC fibril- (**D**) isolated IVIg IgGs against plate-immobilized LC fibrils. Each data point represents the average value from three binding studies, which were carried out using assay buffer consisting of 1% BSA in PBS containing 0.05% tween 20, pH 7.4. (**E**) The % IgGs isolated for each of four sequential passages of a preparation of 10 mg/ml IVIg in PBS, pH 7.4, through a column consisting of Aβ or LC (○) fibrils covalently cross-linked to N-hydroxysuccinimide (NHS) Sepharose®4 fast-flow agarose matrix (Amersham Biosciences Corp.). The plots also show the % IgGs isolated from IVIg over a control unconjugated deactivated sepharose matrix (beads only). The concentration of antibody in eluant preparations was determined by absorbance at 280 nm using an extinction coefficient of 1.25 and a relative molecular mass of 150,000. (**F**) The % depletion of anti-amyloidogenic NAbs in IVIg flow through preparations from Aβ and LC fibril columns, respectively, compared to the unfractionated preparation. Each antibody flow through preparation was generated from the fourth passage of IVIg through a fibril column, as described in Panel E (IgG eluant and flow through was generated for each passage). The % depletion of Aβ conformer and LC fibril reactive NAbs in each antibody flow through was determined from the absorbances at 280 nm of antibody eluants generated by passing IVIg and the antibody flow through preparations through Aβ conformer or LC fibril columns.

NAb’s preference for conformational or linear sequence epitopes on Aβ was determined by ELISA and affinity column chromatography using as substrates, Aβ conformers (monomers, CAPS, fibrils) and non-Aβ fibrils that consisted of LC protein with no known primary sequence identity with Aβ. The antibody binding curves and column depletion studies shown in Figure 1 demonstrate that the majority, if not all, of NAb’s binding to Aβ was directed against conformational epitope(s) that were similarly exposed on LC fibrils. In particular, NAbs isolated from IVIg after one passage through an Aβ or LC fibril column recognized either fibril species, but had a slight (∼3-fold) preference for the plate-immobilized fibril that the antibodies were purified against, with antibody concentration at half maximal effect, EC_50,_ of ∼40 nM (Fig. 1A–D, Table 1). In contrast, anti-Aβ NAbs isolated and pooled from four sequential passages of an IVIg preparation through either fibril column (eluant and flow through generated for each passage) had similar reactivity with plate-immobilized Aβ fibrils and LC fibrils, with EC_50_ values of ∼60 nM. The antibody flow through from the fourth passage for each column was similarly depleted (∼80–100%) against binding to Aβ conformers (monomers, CAPS, fibrils) and LC fibril column matrices, even though the preparations still contained ∼99% of antibodies that were originally applied to the columns (Fig. 1E–F). SDS-PAGE analysis confirmed that the antibody eluants had no detectable Aβ or LC that may have leaked off the affinity matrices.

**Table 1:** Unmodified and Aβ and LC fibril-isolated NAbs binding to plate-immobilized amyloid fibrils.

	Aβ fibrils		LC fibrils		
IVIg	EC_50_ 1	Max signal^1^	EC_50_	Max signal	EC_50_ Aβ fibrils
	(nM)	Eu^+2^ (fm)	(nM)	Eu^+2^ (fm)	EC_50_ LC fibrils
Unfractionated	1012±74	146±3.4	2000±24	92±6.5	0.50
Aβ fibril enriched	38±0.2	263±5.3	137±0.9	147±5.4	0.28
Aβ fibril residual	∼3000	>100	∼5000	>90	–
LC fibril enriched	122±0.7	215±6.8	52±0.1	175±1.8	2.3
LC fibril residual	2884±11	133±2.4	3251±58	77±8.6	0.89

1 Each value for EC_50_ and max signal for NAb binding to plate-immobilized Aβ or LC fibrils was the average value obtained from three sigmoidal fitted antibody binding curves, such as shown in Figure 1.

**Figure 2:**
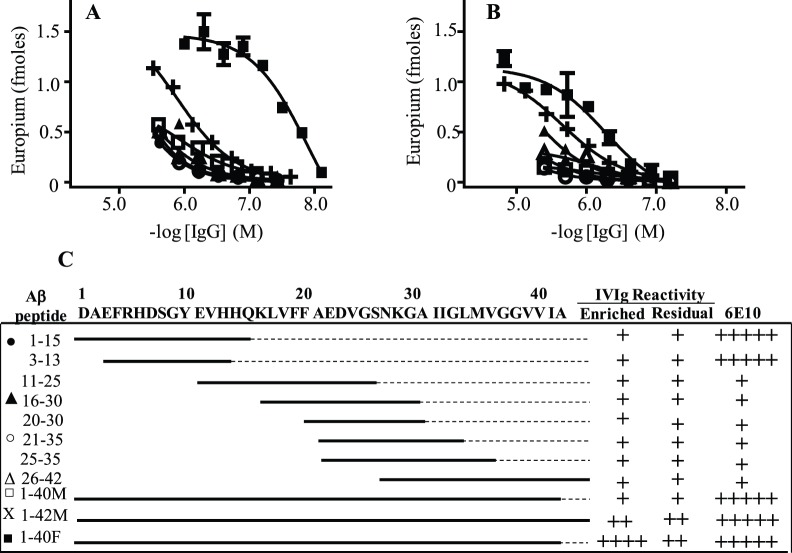
NAbs binding to Aβ conformers and overlapping peptide fragments. The antibody binding curves are representative of Aβ fibril-isolated (**A**) and column flow through (depleted) (**B**) IVIg fractions against plate-immobilized Aβ conformers and overlapping Aβ peptide fragments, respectively. The data symbols represent antibody binding to the Aβ peptides specified in Panel C. (**C**) Schematic comparison of Aβ fibril-isolated and flow through IVIg fractions, as well as an anti-Aβ N-terminal mAb, 6E10, binding to the plate-immobilized Aβ conformers and overlapping peptide fragments.

The NAb’s reliance on Aβ’s conformational state, and not a particular linear sequence segment, was confirmed by Aβ fibril-isolated Nab’s nM binding to plate-immobilized Aβ fibrils, and only weak µM reactivity with monomers and overlapping peptide fragments of the 40- and 42-mer Aβ peptides (Fig. 2). The anti-Aβ NAbs bound ∼40-fold stronger to plate-immobilized Aβ fibrils than control NAbs from an Aβ fibril column flow through preparation. Notably, both the anti-Aβ NAb and antibody flow through preparations had similar µM avidity for plate-immobilized Aβ monomers and peptide fragments, indicating that these interactions were nonspecific. The NAb’s relatively weak interactions with Aβ monomers was not due to an antibody-bound antigen masking Aβ reactivity since pretreating the NAb’s to dissociate such complexes, using a low pH (3.5) method [36], only enhanced antibody reactivity ∼2-fold against plate-immobilized Aβ monomers (Fig. S4). Moreover, the antibodies had enhanced binding to the ELISA blocking agent, BSA, compared to untreated NAbs, indicating that acid pretreatment induced non-specific IgG polyreactivity. In contrast to NAbs, a control anti-Aβ N-terminal mAb, 6E10, bound similarly to plate-immobilized Aβ fibrils, and N-terminal Aβ fragments, Aβ(1–15) and Aβ(3–13) (Fig. 2).

**Figure 3:**
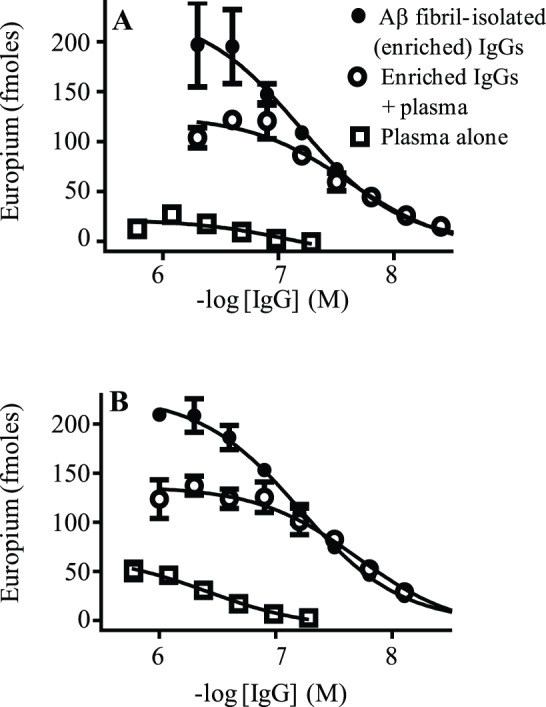
Plasma inhibition of NAbs binding to Aβ conformers. The antibody binding curves show Aβ fibril-isolated IVIg IgGs binding to plate-immobilized Aβ fibrils (**A**) and CAPS (**B**) in the absence or presence of a 1∶10 dilution of normal human plasma. The plots also show Aβ conformer binding by plasma IgGs alone. The data symbols in Panels A and B represent Aβ conformer binding by antibody preparations as specified in the legend of Panel A.

Given the Nab’s propensity to cross-react with Aβ and LC aggregates [17], we established if these antibody’s still maintained binding to Aβ conformers in the presence of normal human plasma and CSF – bodily fluids which contain endogenous molecules that bind to amyloidogenic assemblies [37]. Figure 3 and Table 2 show that normal human plasma had only a modest effect on NAb binding to plate-immobilized CAPS and Aβ fibrils. Plasma reduced the maximum binding signal amplitude by ∼40%, but did not affect strong antibody-fibril interactions since at relatively low antibody concentrations, depicted by the sloping segments of the binding curves, the fluid had no effect. Plasma’s net effect was to lower EC_50_ values for NAbs binding to plate-immobilized CAPS and Aβ fibrils by ∼3-fold, with EC_50_ values of ∼30 nM (Fig. 3, Table 2). In contrast, CSF had no effect on NAb binding to Aβ (data not shown).

**Table 2:** Plasma inhibition of NAbs binding to Aβ conformers.

	No plasma		+ Plasma		Plasma alone	
Aβ conformer	EC_50_ 1	Max signal^1^	EC_50_	Max signal	EC_50_	Max signal
	(nM)	Eu^+2^ (fm)	(nM)	Eu^+2^ (fm)	(nM)	Eu^+2^ (fm)
Fibrils	67±1	232±15	26±0.4	121±10	350±0.6	26±4
CAPS	62±0.2	263±5.3	21±0.1	132±4.0	341±1.5	54±2

1 Each value for EC_50_ and max signal for anti-Aβ NAbs, isolated from IVIg using Aβ fibril affinity chromatography, binding to plate-immobilized Aβ fibrils and CAPS was the average value obtained from three sigmoidal fitted antibody binding curves, such as shown in Figure 3.

### Anti-Aβ NAbs can be Isolated by Cibacron Blue Affinity Chromatography

**Figure 4:**
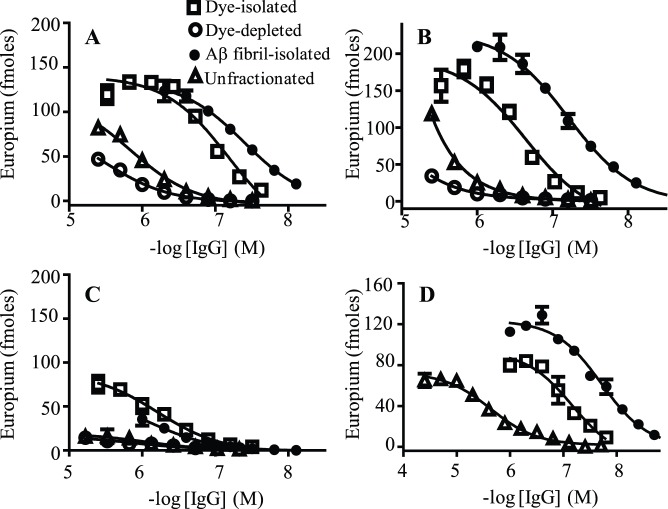
Cibacron blue and Aβ fibril fractionated NAbs binding to amyloidogenic conformers. Antibody binding curves were generated for unfractionated IVIg, Cibacron blue-isolated and depleted, and for Aβ fibril-isolated IVIg IgGs against plate-immobilized Aβ fibrils (**A**), CAPS (**B**), Aβ monomers (**C**), And LC fibrils (**D**). The data symbols in Panels A to D represent Aβ conformer binding by antibody preparations as specified in the legend of Panel A.

Pursuant to using Cibacron blue F3GA to remove albumin from plasma, we discovered that under high salt conditions, anti-Aβ NAbs bound specifically to this dye. Figure 4 and Table 3 show that anti-Aβ NAbs isolated by passing IVIg through a Cibacron blue column bound ∼20-fold stronger to plate-immobilized Aβ fibrils and CAPS than the unfractionated preparation, with EC_50_s of ∼100 nM. In contrast, the same preparation was only ∼2-fold enriched in binding to plate-immobilized Aβ monomers, with EC_50_s of ∼500 nM (Fig. 4, Table 3). Essentially no anti-Aβ IgGs were isolated using the bead matrix alone, confirming that the antibodies bound specifically to the dye. The relatively high ionic strength required to facilitate anti-Aβ NAbs binding to Cibacron blue suggests that dye-NAb complexes were stabilized by hydrophobic interactions. However, we were not able to specifically isolate anti-Aβ IgGs from IVIg using a standard hydrophobic matrix, phenyl sepharose CL-4B (Sigma). The amount of antibody that eluted off the dye column after one passage, determined by absorbance at 280 nm, corresponded to ∼0.6% of the total IgGs applied, and was ∼3-fold more than was obtained using a similarly sized Aβ fibril column. The relatively large amount of dye-isolated antibodies presumably reflected a greater proportion of Aβ-unreactive antibodies than was present in Aβ fibril-isolated antibody preparations, and resulted in a ∼2-fold larger EC_50_ value of ∼100 nm for dye-isolated NAbs binding to plate-immobilized Aβ aggregates (Fig. 4, Table 3). Despite the latter differences, two experimental findings indicate that the dye- and fibril-isolated antibody preparations contained similar anti-Aβ IgGs. First, four sequential passages of an IVIg preparation through either the dye or fibril column (eluant and flow through generated for each passage) removed ∼1% of the antibodies that were originally applied to the column, but the final antibody flow through was >90% reduced in binding to an Aβ fibril column. Lastly, both the dye- and Aβ fibril-isolated IgGs were similarly cross-reactive with Aβ and LC fibrils (Fig. 4D, Table 3).

**Table 3:** Unfractionated, Cibacron blue- and Aβ fibril-isolated NAbs binding to plate-immobilized amyloidogenic conformers.

	Unfractionated		Cibacron blue-isolated		Aβ Fibril-isolated	
Conformer	EC_50_ 1	Max signal	EC_50_	Max signal	EC_50_	Max signal
	(nM)	Eu^+2^ (fm)	EC_50_	Eu^+2^ (fm)	(nM)	Eu^+2^ (fm)
Aβ Fibrils	927±5	89±4	105±1	131±8	45±1	133±4
CAPS	∼3000	>100	120±2	168±6	62±0.3	226±4
Aβ Monomers	∼1000	>15	542±4	82±1	547±1	106±2
LC fibrils	2160±20	35±2	46±1	43±2	20±1	61±3

1 Each value for EC_50_ and max signal for Aβ-reactive NAbs binding to a plate-immobilized amyloidogenic conformer was determined from the average value obtained from three sigmoidal fitted antibody binding curves, such as shown in Figure 4.

### NAbs Recognize Synthetic Aβ Dimer Assemblies and AD Brain-derived Aβ

An array of Aβ assemblies are likely to mediate toxicity, but burgeoning evidence suggests that water-soluble non-fibrillar assemblies, including those formed from Aβ dimers, have disease-relevant activity [4], [25], [30], [38]. Consequently, we investigated NAbs ability to bind to soluble Aβ dimer assemblies by ELISA and immunoprecipitation/Western blot analyses using synthetic [S26CAβ]_2_ and synaptotoxic PFs formed from [S26CAβ]_2_ [30], [38]. Anti-Aβ NAbs used in these studies were isolated by Cibacron blue and not by fibril affinity chromatography since a very small amount of Aβ was identified in Aβ fibril-isolated antibody preparations (∼0.2 µg/ml Aβ (46 nM w.r.t. monomers) in ∼2 mg/ml antibody samples) using a highly sensitive immunoprecipitation/Western blot protocol [34]. The contaminating peptide was only sufficient to bind to ∼0.4% of the fibril-isolated NAbs (assuming 1∶1 interactions of Aβ with IgG), and did not sufficiently affect ELISA determination of antibody avidity, but was sufficient to compromise immunoprecipiation/Western blot investigations.

**Figure 5:**
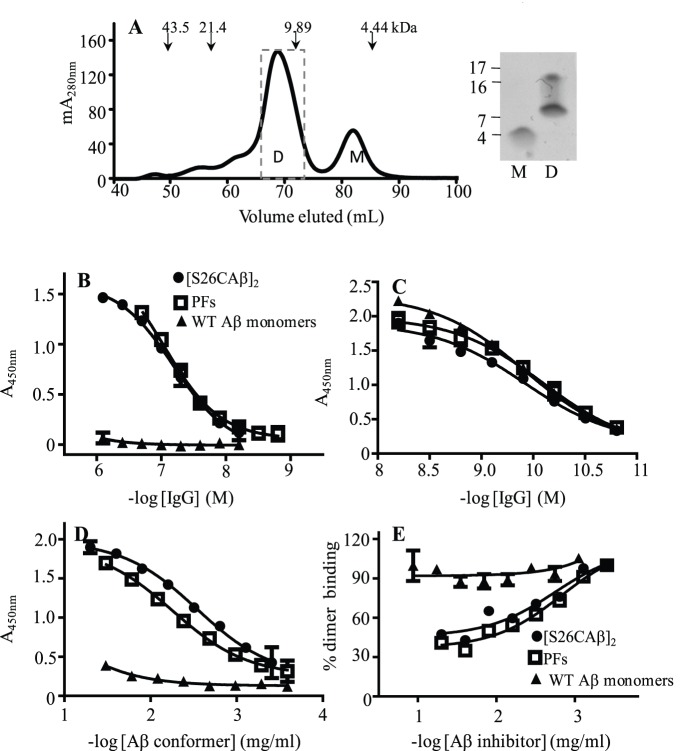
Isolation of Aβ dimers and NAb binding. (**A**) [S26CAβ]_2_ was isolated by SEC using a HiLoad 16/60 Superdex 75 column equilibrated with 25 mM ammonium acetate, pH 8.5. Arrows indicate elution of linear dextran standards, and D and M are abbreviations for Aβ dimers and monomers, respectively. SDS-PAGE analysis of the low molecular weight SEC peaks confirmed the presence of dimers or monomers. A portion of Aβ in the dimer fraction migrated as an ∼16 kDa assembly that was transiently and artificially induced by the detergent [30], [39] (**B**) Cibacron blue-isolated IVIg IgGs, (**C**) anti-Aβ N-terminal mAb, 6E10, binding to plate-immobilized Aβ conformers: [S26CAβ]_2_; PFs, and WT Aβ monomers. (**D**) Plate-immobilized Cibacron blue-isolated IVIg IgGs binding to solution-phase Aβ conformers, and (**E**) Aβ competition curves for solution-phase Aβ conformer inhibition of 100 nM Cibacron blue-isolated IVIg IgGs binding to plate-immobilized [S26CAβ]_2_. The data symbols in Panels B to E represent antibody binding to Aβ conformers as specified in the legend of Panel B.

The SEC chromatograph shown in Figure 5 indicates that freshly-isolated [S26CAβ]_2_ were highly pure, and based on the use of linear dextran standards, had an apparent molecular weight of ∼10 kDa. A portion of the conformer migrated as a ∼16 kDa assembly in denaturing SDS-PAGE, but this was transiently and artificially induced by the detergent [30], [39]. The dye-isolated NAbs bound >10-fold stronger to plate-immobilized [S26CAβ]_2_ and PFs than to WT Aβ monomers, with EC_50_s of ∼50–100 nM (Fig. 5, Table 4). In contrast, mAb, 6E10 did not have a preference for any of the plate-immobilized Aβ conformers (Table 4).

**Table 4:** Unfractionated and Cibacron blue-isolated NAbs binding to plate-immobilized amyloidogenic conformers.

	Direct ELISA				CB^1^-isolated IVIg		
	CB-isolated IVIg		mAb 6E10		Aβ capture		Aβ comp
Aβ Conformer	EC_50_ 2	Max^3^	EC_50_	Max	EC_50_	Max	IC_50_ 2
	(nM)	A_450nm_	(nM)	A_450nm_	(µg/ml)	A_450nm_	(µg/ml)
[S26CAβ]_2_ PFs	64±2	1.6±0.1	0.12±0.01	2.0±0.1	4.4±0.02	1.9±0.01	4.4±0.02
[S26CAβ]_2_ dimers	105±2	∼1.50	0.09±0.01	2.1±0.1	9.9±0.3	1.9±0.02	16±0.01
CAPS	50±1	1.3±0.1	0.15±0.02	2.2±0.02	12±0.3	1.6±0.01	>50
WT monomers	>800	–	0.12±0.01	2.4±0.01	>100	–	>100

1 CB is an abbreviation for Cibacron blue.

2 Each value for EC_50_, max signal, and IC_50_ for antibody binding to a plate-immobilized Aβ conformer was the average value obtained from three sigmoidal fitted antibody binding curves, such as shown in Figure 5.

3 Max is an abbreviation for maximum assay signal.

**Figure 6:**
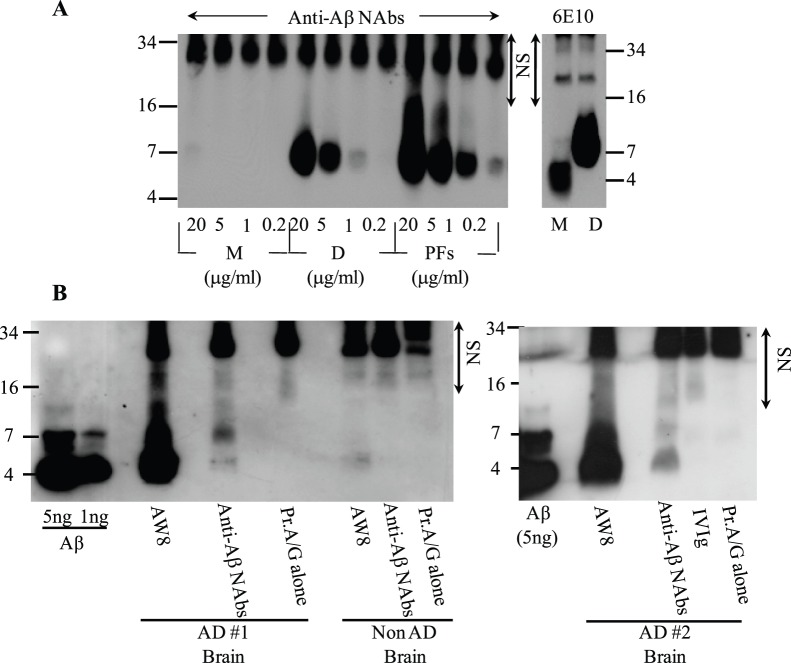
NAb immunoprecipitation of synthetic and AD brain-derived Aβ. (**A**) Western blot analysis of synthetic S26CAβ conformers immunoprecipitated by NAbs isolated from IVIg by Cibacron blue affinity chromatography, and by mAb 6E10: M, monomers; D, dimers, and PFs. (**B**) Western blot analysis of soluble Aβ present in TBS extracts of AD brain immunoprecipitated by unfractionated and Cibacron blue-isolated IVIg IgGs, and by rabbit polyclonal anti-Aβ IgGs, AW8. Prot A/G alone stands for control studies carried out with antibody capture beads (a 1∶1 mixture of Protein A sepharose and Protein G agarose) and brain extract without primary antibody. The Western blots were developed using N-terminal and mid-region Aβ-reactive mAbs, 6E10 and 4G8, and enhanced chemiluminescence as the detection system. NS, indicates non-specific bands arising from secondary antibody detection of NAb’s light chains. M and D are abbreviations for Aβ monomers and dimers, respectively.

Since surface-adsorption of Aβ can artificially induce amyloid-like epitopes [11], [40], we also tested if the Nabs could bind to [S26CAβ]_2_ and PFs in solution. Panel E of Figure 5 and Table 4 show that plate-immobilized anti-Aβ NAbs captured solution-phase [S26CAβ]_2_ and PFs, with EC_50_ values of ∼5–10 µg/ml (0.6–1.2 µM w.r.t. the dimeric peptide). In contrast, the antibodies bound much weaker to WT Aβ monomers (EC_50_>100 µg/ml). NAb’s preference for aggregated Aβ was not an artifact induced by the secondary anti-Aβ polyclonal antibody, AW8, used in the capture ELISA since AW8 bound similarly to saturating amounts of PFs or WT monomers bound to plate-immobilized anti-Aβ NAbs (data not shown). Solution-phase competition ELISA studies, where both NAb and Aβ conformers were in solution, further verified the antibody’s preference for aggregated Aβ. Figure 5F and Table 4 show that [S26CAβ]_2_ and PFs dose-dependently inhibited antibody binding to plate-immobilized [S26CAβ]_2_, with inhibitor concentrations at half maximal effect, IC_50_ values, of ∼4 and 16 µg/ml (∼0.5 and 1.8 µM w.r.t. the dimeric peptide), respectively. In contrast, WT Aβ monomers lacked significant inhibitory activity, with IC_50_ values >50 µg/ml (>11.6 µM) (Fig. 5, Table 4). To verify NAb binding to Aβ conformers in solution, we tested their ability to immunoprecipitate various synthetic Aβ assemblies. Consistent with our ELISA results, Figure 6A shows that the NAbs preferentially bound to [S26CAβ]_2_ and PFs without detectable binding to the monomeric peptide. In contrast mAb 6E10 similarly immunoprecipitated all Aβ conformers (Fig. 6A). Control experiments confirmed that the Aβ aggregates did not significantly bind to the protein A/G matrix alone.

Having demonstrated nM binding by NAbs to synthetic Aβ dimer assemblies, we established if they could recognize Aβ from the water-soluble phase of AD brain [34]. Figure 6B shows that the anti-Aβ NAbs and our control polyclonal anti-Aβ antibody, AW8, immunoprecipitated Aβ (<1 and >5 ng/ml, respectively) that was present in TBS extracts of two AD brains, but had no reactivity with TBS extract of a normal age-matched control brain. Control experiments confirmed that the antibodies and not protein A/G beads alone bound to AD brain-derived Aβ (Fig. 6B). The immunoprecipitated Aβ peptide migrated in denaturing SDS-PAGE as monomers and dimers. Whether the immunoprecipitated Aβ actually existed in solution under native conditions as monomers and dimers is uncertain since we have previously shown that Aβ derived from human brain elutes from SEC with a range of molecular weights, but that when subjected to SDS-PAGE only monomers and dimers are detected [38]. We take these results to mean that Aβ from the aqueous phase of human brain can exist in different sized assemblies, built up of Aβ monomer and/or SDS-stable Aβ dimer (for discussion see [30]), and that the NAbs did not recognize authentic Aβ monomers, but larger Aβ assemblies.

## Discussion

Anti-Aβ immunotherapy is a promising approach for AD, and increasing experimental evidence indicates that soluble Aβ aggregates, including assemblies formed from dimers, are the primary toxic species [5], [6], [26], [30], [41]. IVIg, which contains anti-Aβ NAbs, has shown promise in recent clinical trials for AD, but its mode of action is not yet known [12], [13]. Nevertheless, current research is primarily focused on anti-Aβ NAbs since extensive *in vitro* and transgenic animal studies have indicated that these antibodies have unique therapeutic potential for AD [11], [12], [16], [17], [18], [19], [20]. Our finding that anti-Aβ NAbs have nM avidity for solution-phase synthetic Aβ dimer assemblies and AD brain-derived Aβ, as well as their ability to recognize soluble synthetic cross-linked β-amyloid protein species (CAPS) [11] and Aβ trimers [18], indicates that these antibodies are, at least in part, responsible for IVIg’s modulation of AD patient’s levels of cerebral and blood pool soluble Aβ [12], [13], [18]. In particular, infusions of IVIg have reduced patient’s cerebral Aβ and concurrently increased Aβ’s blood pool – a process consistent with the beneficial effects of anti-Aβ immunotherapy [5], [6]. Notably, Dodel’s lab have recently shown that vaccinating AD transgenic mice with anti-Aβ NAbs, which were isolated from IVIg using an Aβ affinity column, reduced cerebral Aβ levels, increased the peptide’s blood pool, and significantly improved the animal’s object location memory [18].

Anti-Aβ NAb-mediated clearance of cerebral Aβ may occur through several, not mutually exclusive, mechanisms. This includes Fc-mediated clearance, rapid efflux of Aβ to the periphery via antibody-Aβ complexes, antibody inhibition of Aβ aggregation, and a peripheral sink mechanism whereby antibody targeting of blood pool Aβ alters an equilibrium between brain and blood pool Aβ that causes an efflux of the peptide into the periphery [42], [43]. Our findings suggest that the latter peripheral sink mechanism is the most plausible mode of action for anti-Aβ NAbs since their modest (nM) avidity for solution-phase Aβ aggregates may be offset by their relatively high concentration in the blood. In contrast, NAb binding to cerebral Aβ is likely to be minimal since only ∼0.1% of these antibodies may enter the brain [44]. Furthermore, our demonstration that NAbs retain Aβ binding in plasma indicates that they are not significantly inhibited from reacting with peripheral Aβ by endogenous plasma molecules [37]. Alternatively, IVIg’s modulation of Aβ in bodily fluids of AD patient and transgenic mice may not be due to NAb’s modulation of an equilibrium between cerebral and blood pool Aβ, but their direct clearance of Aβ in the brain, and peripheral NAbs binding to blood pool Aβ, which in itself can increase the peptide’s half-life [42], [45]. Levels of cerebral Aβ may have also been reduced by NAbs in IVIg that can target a receptor for advanced glycation end products (RAGE) [46], a protein that transports Aβ into the brain [47], and/or soluble low-density lipoprotein receptor-related protein-1 (sLRP1) [48], a major Aβ binding protein in the blood that is present in IVIg [46].

The proximate pathogenic Aβ specie(s) to target are still unknown, but assemblies formed from Aβ dimers are prime candidates [26], [30], [33], [38]. The NAbs nM avidity for synthetic Aβ dimer assemblies, [S26CAβ]_2_ and synaptotoxic PFs, and AD brain-derived Aβ suggests that these antibodies recognize the pathogenic peptide [30], [38]. As mentioned above, although NAbs can immunoprecipitate AD brain-derived Aβ, their modest avidity for these conformers, and inability to penetrate the blood brain barrier, suggests that these antibodies are more likely to target Aβ in the periphery, such as plasma membrane-bound Aβ dimers [27]. Nevertheless, anti-Aβ antibodies that enter the brain may still benefit an AD patient by clearing Aβ to the periphery, and/or by neutralizing toxic Aβ species [8], [16], [18], [21] (Fig. S5).

The conformational Aβ epitope(s) recognized by NAbs presumably consist of sequentially discontinuous segments that are close together in three-dimensional space [49]. Our observation that these surfaces are similarly exposed on Aβ aggregates and LC fibrils is consistent with our previous observations that NAbs can react with aggregated Aβ and are pan amyloid fibril-reactive [11], [17]. Such epitopes may be formed from Aβ-Aβ interactions, or could also be generated *in vivo* from the interaction of monomeric peptide with endogenous molecules or matrices [11], [40]. The inability of NAbs to recognize synthetic Aβ monomers or the fragmented peptide contrasts with previous studies that suggested anti-Aβ NAbs specifically recognize linear sequence segments (for example [18], [19], [50]). However, the latter studies primarily involved surface-immobilized synthetic Aβ, which due to surface adsorption can artificially expose amyloidogenic epitopes that are not present on the solution-phase native peptide [11]. Also, NAb binding to Aβ was not compared to control antibodies, such as anti-Aβ depleted IVIg, nor was the antibody’s reactivity established against Aβ monomers and aggregates. To address these anomalies, we investigated anti-Aβ and control NAbs dose-dependent binding to plate-immobilized Aβ monomers, fibrils, and overlapping Aβ fragments. Our investigations clearly showed that although anti-Aβ NAbs bound to plate-immobilized Aβ monomers and peptide fragments, this reactivity was very weak, in the µM range, and non-specific since similar binding was obtained with the control Nabs. Furthermore, the anti-Aβ NAbs bound ∼40-fold more avidly to plate-immobilized Aβ fibrils than the control antibody, confirming that strong Nab-Aβ interactions were critically dependent on the peptide’s conformational state.

Natural human antibodies make up about a third of an individual’s antibody repertoire, target a wide spectrum of self and non-self antigens, and have been implicated in disease and a plethora of physiological functions [51]. Although the function and molecular basis for anti-Aβ NAbs is not yet known, we have demonstrated their specificity for conformational epitope(s) on Aβ aggregates, dimers to fibrils, and LC fibrils. We have also previously demonstrated that anti-Aβ NAb’s are inherently present in normal, presumably healthy individuals, are pan-amyloid fibril reactive, cross-react with CAPS, and have therapeutic potential [11], [17], [52]. The NAb’s ability to cross-react with a plethora of amyloidogenic assemblies suggests that these IgGs may have evolved to neutralize and/or clear endogenous misfolded proteins containing amyloid-like epitopes in the chaperone-free intercellular milieu. Such conformational binding surfaces presumably contain unique clusters of hydrogen bond donor/acceptor groups, solvent exposed amino acid side chains, and/or a unique chain reversal that are not exposed on natively folded polypeptides [23]. Our ability to isolate anti-Aβ NAbs from IVIg in high salt buffer using Cibacron blue affinity chromatography indicates that these molecules bind a common, limited set of epitope(s) that have a hydrophobic component. However, the molecular composition of these epitope(s) remains elusive since we were unable to isolate anti-Aβ NAbs using a standard hydrophobic matrix, phenyl sepharose CL-4B (Sigma).

In summary, we have demonstrated that anti-Aβ NAbs primarily target conformational epitope(s) on soluble synthetic Aβ assemblies, dimers to fibrils, AD brain-derived Aβ, and these surfaces are similarly exposed on LC fibrils. The latter findings indicate that further investigations on the molecular basis and therapeutic/diagnostic potential of anti-Aβ NAbs are warranted. Although IVIg is limited in supply and there may not be enough to treat the AD patient population, advancing understanding on the molecular basis for NAb-Aβ interactions should facilitate the generation of a more renewable therapeutic reagent, such as human monoclonal anti-Aβ NAbs.

## Supporting Information

Figure S1 **Quiescent aggregation of [S26CAβ]_2_**. (**A**) Progress curves for the formation of ThT positive material from 22 µM [S26CAβ]_2_ (•) and S26CAβ monomers (○). The reaction was carried out at 37°C in 40 mM sodium phosphate, pH 7.4. Negative contrast EM was performed on freshly SEC-isolated [S26CAβ]_2_ (**B**), and on the aggregation reaction product (**C**). (**D**) A high magnification of the image shown in panel C. Scale bar, 100 nm.
(TIF)

Figure S2 **Biophysical properties and ThT fluorescence of CAPS and LC fibrils**. Negative contrast EM was performed on 0.2 mg/ml CAPS reaction product (**A**–**B**), and on the aggregation products of Aβ (**C**) and LC (**D**) fibrils formation reactions. (**E**) Relative ThT fluorescent signal for 3 µg Aβ fibrils, LC fibrils CAPS. (**F**) Emission wavelength spectra of 50 µM CAPS (•), and 50 µM of Aβ monomers (○) in PBS, pH 7.4, with excitation at 320 nm.
(TIF)

Figure S3 **ThT fluorescence and circular dichroism spectra for Aβ conformers**. (**A**) Relative ThT fluorescent signal for the same weight (3 µg) of WT and S26C Aβ conformers. (**B**) Circular dichroism spectra obtained for freshly prepared 20 µM [S26CAβ]_2_ (− −), CAPS (–), and WT Aβ monomers (– –) in 25 mM ammonium acetate, pH 8.5.
(TIF)

Figure S4 **Acid-induced enhancement of IVIg binding to plate-immobilized Aβ**. (**A**) Antibody binding curves against plate-immobilized Aβ monomers for IVIg that was unmodified (○), pretreated with acid (0.1 M glycine, pH 3.5), concentrated (Pierce concentrator, 20 kDa m.w.c.o.), and then neutralized with 1 M Tris (pH 9.0) (Δ), or concentrated without treatment with acid/neutralization buffer (•).
(TIF)

Figure S5 **The effect of IVIg on Aβ’s neurotoxicity**. Rat pheochromocytoma PC12 cells (CRL-1721, ATCC) were incubated with 10 µM Aβ1–42 (American Peptide) alone or with 20 mg/ml IVIg. The Aβ1–42 peptide used was solubilized to ∼1 mg/ml in 10 µM Tris/HCl (pH 8.6), sonicated in a water bath for 5 min, and 0.1% HCl added to adjusted the pH to 7.2. PC12 cells (1×10^5^ cells/ml) were cultured in Neurobasal-A medium with 2% B27 serum substitute and 2 mM L-Glutamine (all from Life Technologies) at 37°C and 5% CO_2_. The cells were seeded into 96-well microplates at 100 µl/well and supplemented with 10 µl/well of a 20% human serum albumin solution (Baxter). IVIg (Gammagard Liquid, Baxter) dialyzed in Neurobasal-A medium or Neurobasal-A medium alone was added before the cells were incubated with 10 µM Aβ_42_ in a final volume of 200 µl/well. After a 22 h incubation, 2 µl/well Triton X-100 was added to determine the maximum LDH release and the plate was incubated for additional 2 h. LDH release from the PC12 cells was determined using the LDH Cytotoxicity Detection Kit (Roche) according the manufacturer’s instructions. PC12 toxicity is depicted as optical density (OD). Data represent 6 independent experiments, each performed using 4-8 replicates, and are given as means±SEM. Significant differences between the groups were assessed by one-way ANOVA followed by Tuckey’s post hoc test. *** and ** represent p-values of <0.001 and <0.01, respectively, and n.s. indicate not significant.
(TIF)
