# Supplementary figures and images for: Human Anti-Aβ IgGs Target Conformational Epitopes on Synthetic Dimer Assemblies and the AD Brain-Derived Peptide
Source: PLoS One. 2012 Nov 27;7(11):e50317. doi: 10.1371/journal.pone.0050317 (PMC3507685; doi:10.1371/journal.pone.0050317)

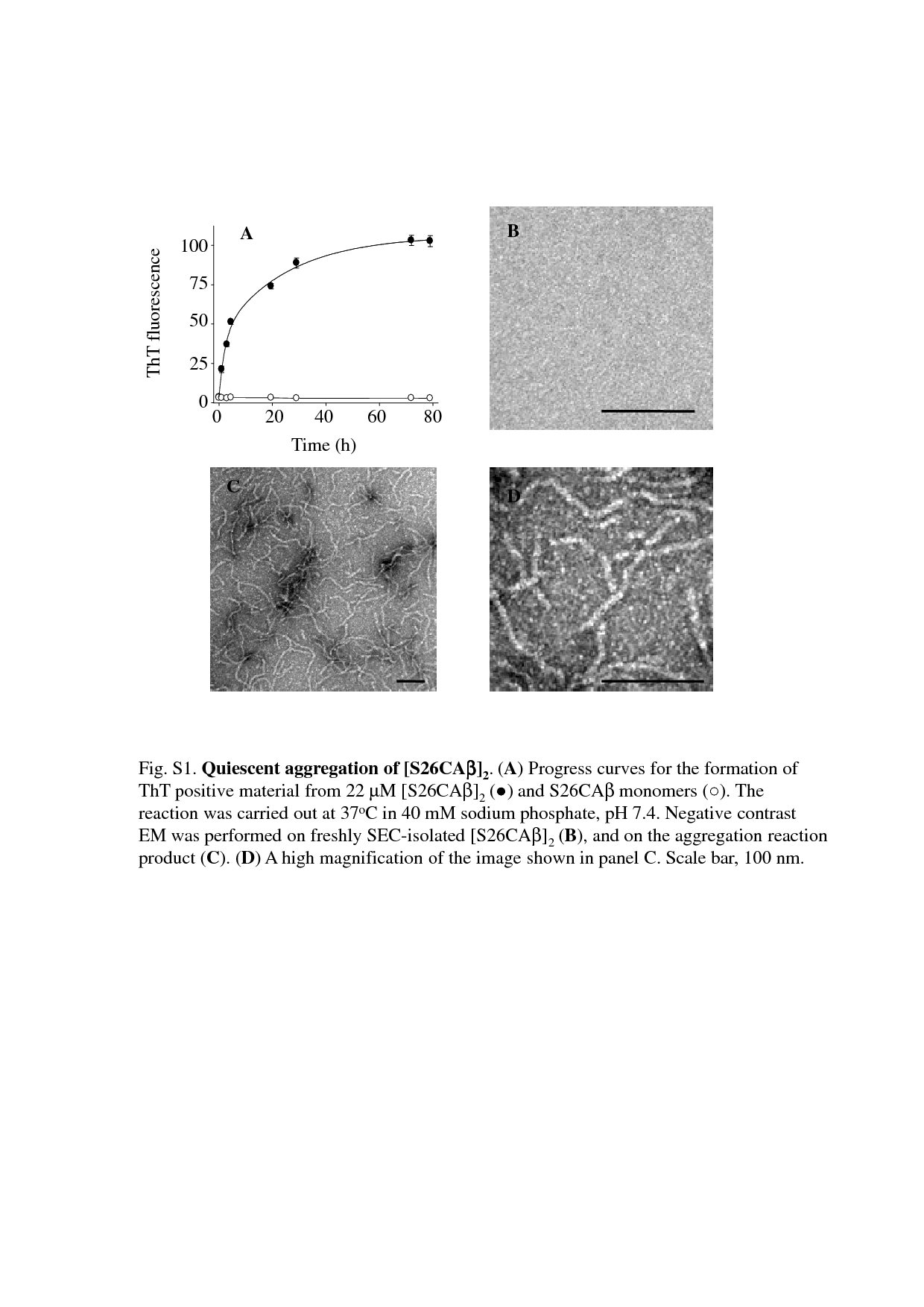

Supplement: Figure S1 — Quiescent aggregation of [S26CAβ]2. (A) Progress curves for the formation of ThT positive material from 22 µM [S26CAβ]2 (•) and S26CAβ monomers (○). The reaction was carried out at 37°C in 40 mM sodium phosphate, pH 7.4. Negative contrast EM was performed on freshly SEC-isolated [S26CAβ]2 (B), and on the aggregation reaction product (C). (D) A high magnification of the image shown in panel C. Scale bar, 100 nm. (TIF) [file pone.0050317.s001.tif]

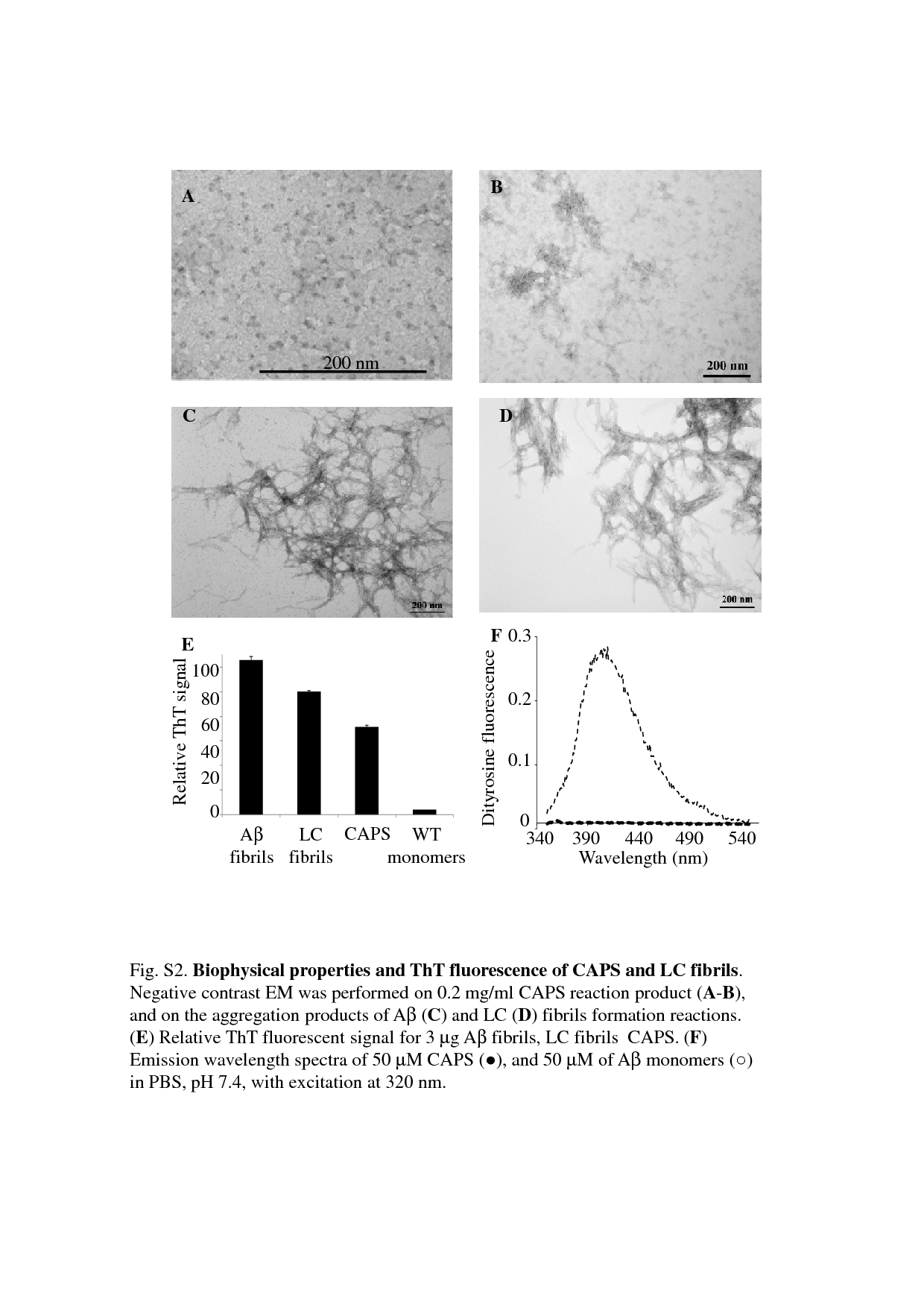

Supplement: Figure S2 — Biophysical properties and ThT fluorescence of CAPS and LC fibrils. Negative contrast EM was performed on 0.2 mg/ml CAPS reaction product (A–B), and on the aggregation products of Aβ (C) and LC (D) fibrils formation reactions. (E) Relative ThT fluorescent signal for 3 µg Aβ fibrils, LC fibrils CAPS. (F) Emission wavelength spectra of 50 µM CAPS (•), and 50 µM of Aβ monomers (○) in PBS, pH 7.4, with excitation at 320 nm. (TIF) [file pone.0050317.s002.tif]

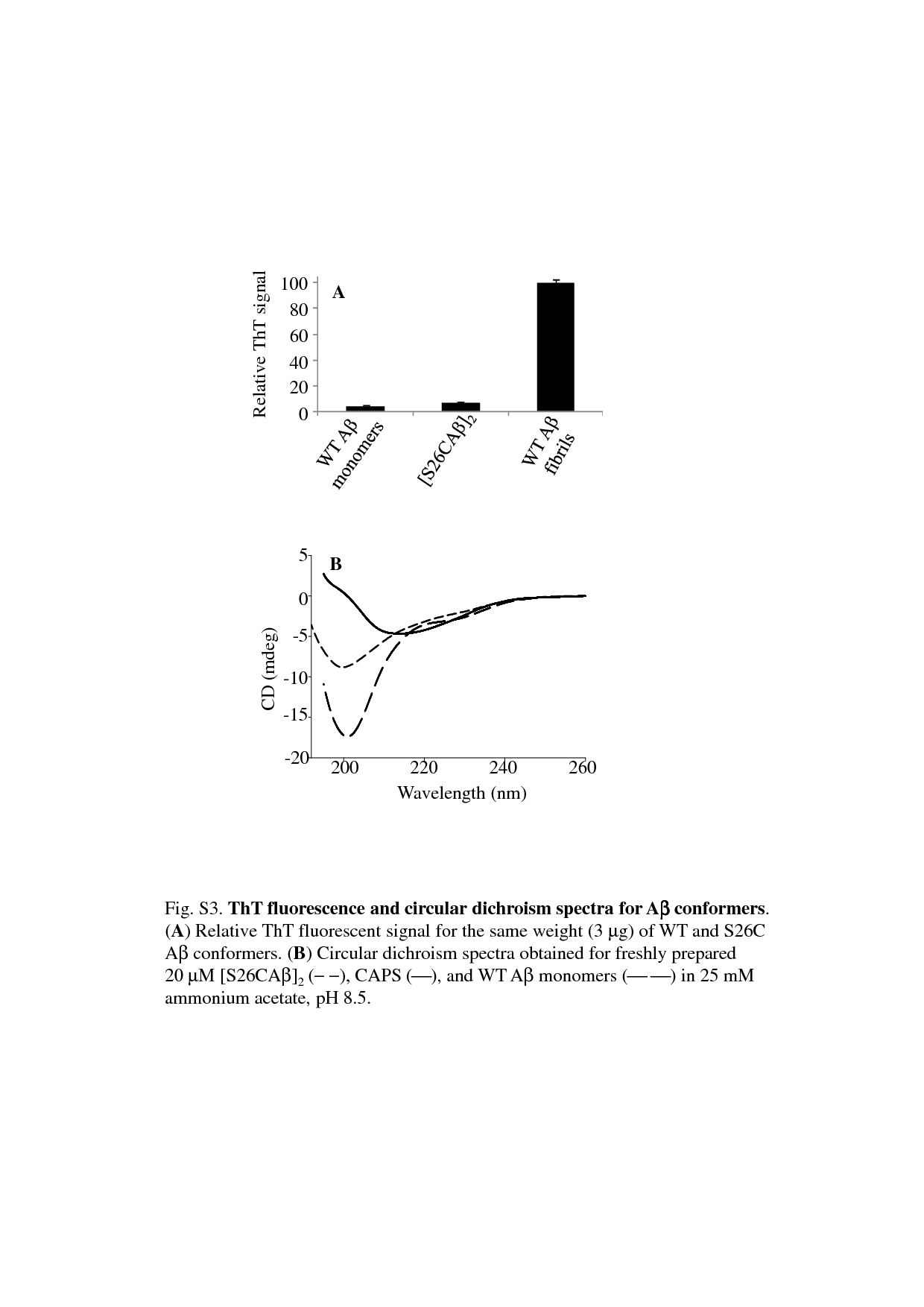

Supplement: Figure S3 — ThT fluorescence and circular dichroism spectra for Aβ conformers. (A) Relative ThT fluorescent signal for the same weight (3 µg) of WT and S26C Aβ conformers. (B) Circular dichroism spectra obtained for freshly prepared 20 µM [S26CAβ]2 (− −), CAPS (–), and WT Aβ monomers (– –) in 25 mM ammonium acetate, pH 8.5. (TIF) [file pone.0050317.s003.tif]

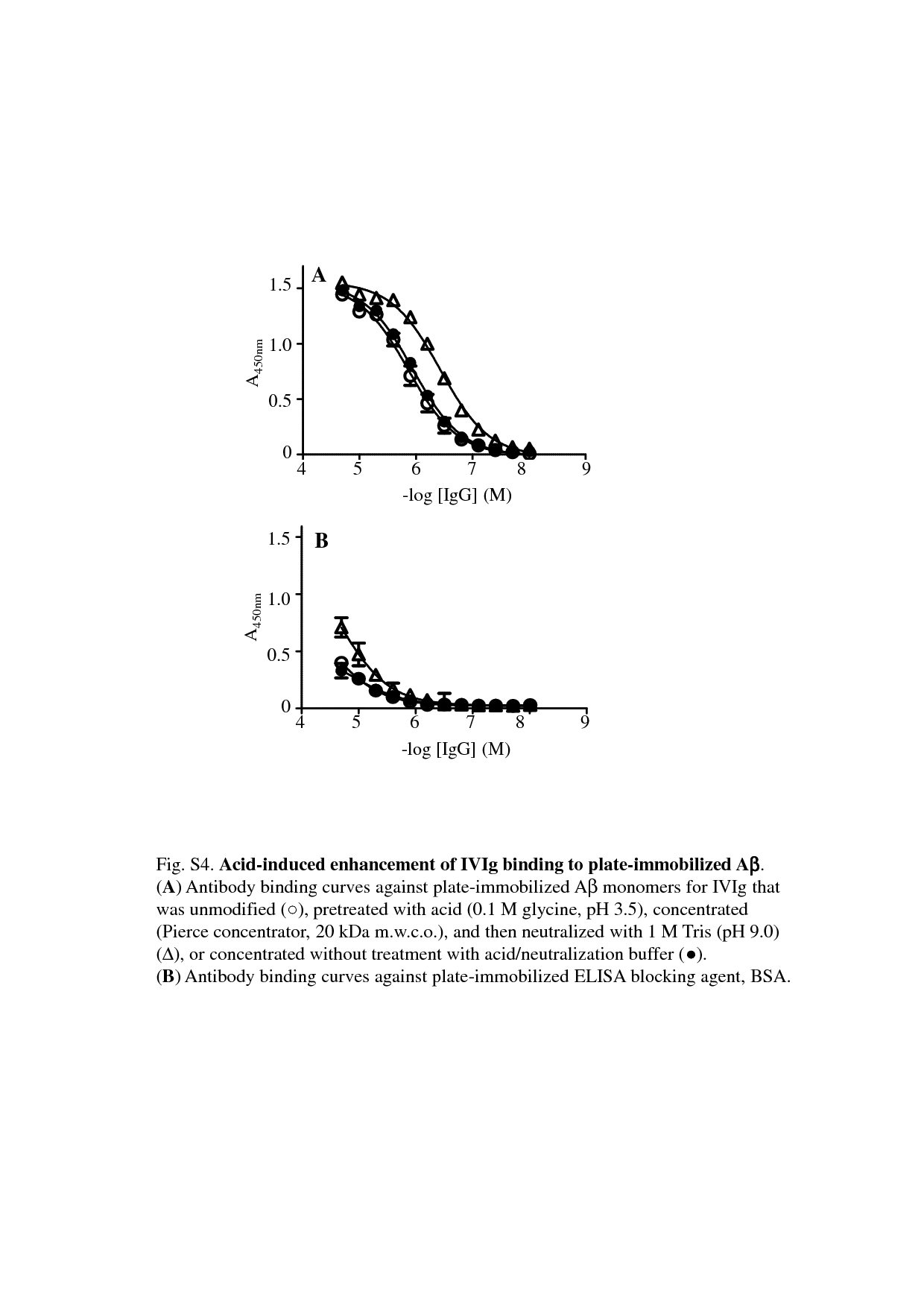

Supplement: Figure S4 — Acid-induced enhancement of IVIg binding to plate-immobilized Aβ. (A) Antibody binding curves against plate-immobilized Aβ monomers for IVIg that was unmodified (○), pretreated with acid (0.1 M glycine, pH 3.5), concentrated (Pierce concentrator, 20 kDa m.w.c.o.), and then neutralized with 1 M Tris (pH 9.0) (Δ), or concentrated without treatment with acid/neutralization buffer (•). (TIF) [file pone.0050317.s004.tif]

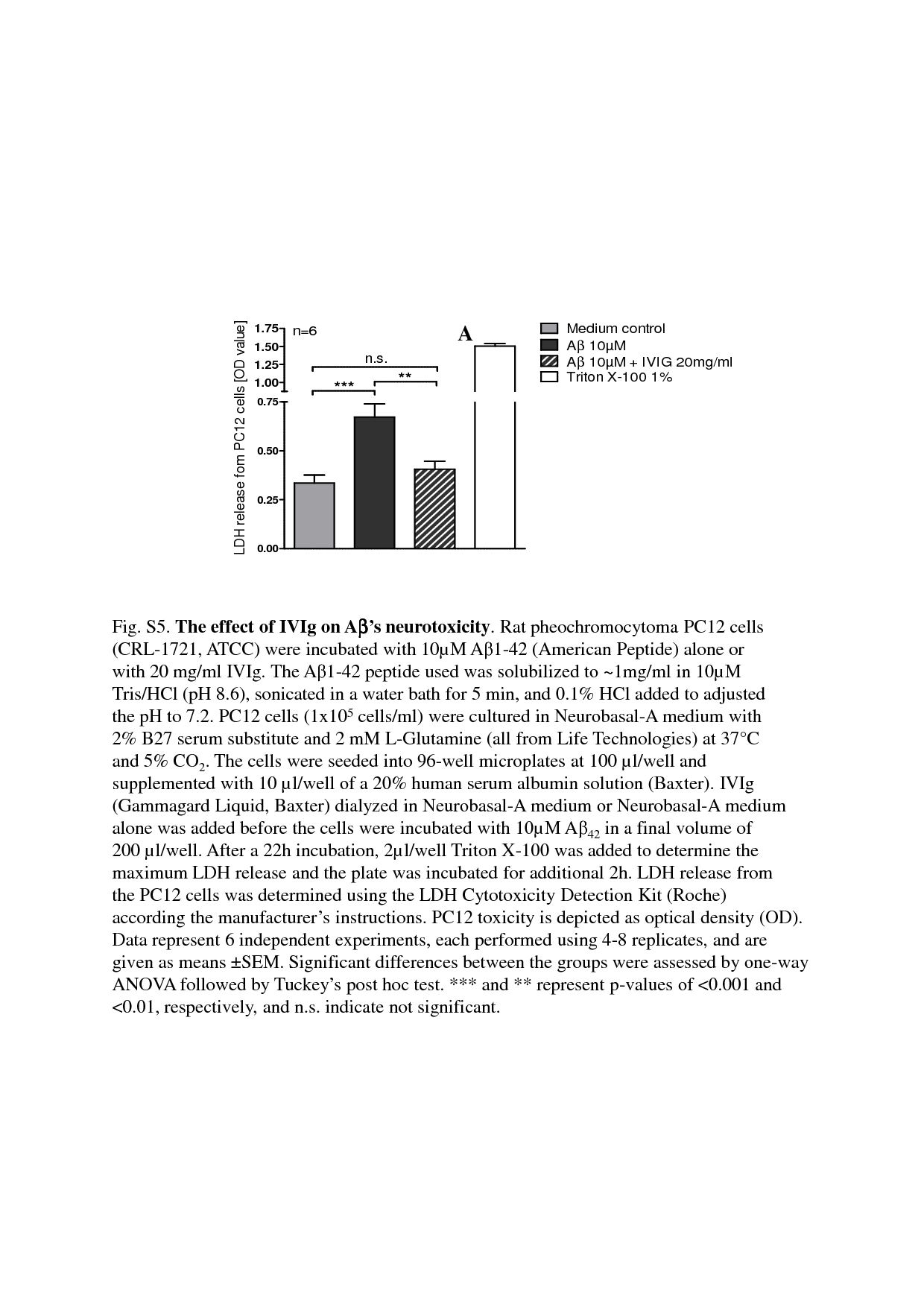

Supplement: Figure S5 — The effect of IVIg on Aβ’s neurotoxicity. Rat pheochromocytoma PC12 cells (CRL-1721, ATCC) were incubated with 10 µM Aβ1–42 (American Peptide) alone or with 20 mg/ml IVIg. The Aβ1–42 peptide used was solubilized to ∼1 mg/ml in 10 µM Tris/HCl (pH 8.6), sonicated in a water bath for 5 min, and 0.1% HCl added to adjusted the pH to 7.2. PC12 cells (1×10^5^ cells/ml) were cultured in Neurobasal-A medium with 2% B27 serum substitute and 2 mM L-Glutamine (all from Life Technologies) at 37°C and 5% CO2. The cells were seeded into 96-well microplates at 100 µl/well and supplemented with 10 µl/well of a 20% human serum albumin solution (Baxter). IVIg (Gammagard Liquid, Baxter) dialyzed in Neurobasal-A medium or Neurobasal-A medium alone was added before the cells were incubated with 10 µM Aβ42 in a final volume of 200 µl/well. After a 22 h incubation, 2 µl/well Triton X-100 was added to determine the maximum LDH release and the plate was incubated for additional 2 h. LDH release from the PC12 cells was determined using the LDH Cytotoxicity Detection Kit (Roche) according the manufacturer’s instructions. PC12 toxicity is depicted as optical density (OD). Data represent 6 independent experiments, each performed using 4-8 replicates, and are given as means±SEM. Significant differences between the groups were assessed by one-way ANOVA followed by Tuckey’s post hoc test. *** and ** represent p-values of <0.001 and <0.01, respectively, and n.s. indicate not significant. (TIF) [file pone.0050317.s005.tif]
